# Microbial alkyl- and aryl-sulfatases: mechanism, occurrence, screening and stereoselectivities

**DOI:** 10.1007/s00253-013-5438-0

**Published:** 2013-12-19

**Authors:** Michael Toesch, Markus Schober, Kurt Faber

**Affiliations:** Department of Chemistry, Organic and Bioorganic Chemistry, University of Graz, Heinrichstrasse 28, 8010 Graz, Austria

**Keywords:** Alkyl sulfatase, Inversion, Retention, Mechanism, Biocatalysis

## Abstract

This review gives an overview on the occurrence of sulfatases in Prokaryota, Eukaryota and Archaea. The mechanism of enzymes acting with retention or inversion of configuration during sulfate ester hydrolysis is discussed taking two complementary examples. Methods for the discovery of novel alkyl sulfatases are described by way of sequence-based search and enzyme induction. A comprehensive list of organisms with their respective substrate scope regarding *prim*- and *sec*-alkyl sulfate esters allows to assess the capabilities and limitations of various biocatalysts employed as whole cell systems or as purified enzymes with respect to their activities and enantioselectivities. Methods for immobilization and selectivity enhancement by addition of metal ions or organic (co)solvents are summarised.

## Introduction — inverting hydrolases

Stereochemical changes, such as inversion of configuration involving a chiral carbon center require mechanistically sophisticated pathways (But and Toy 2007). In nature, several types of hydrolytic enzymes are able to facilitate such reactions by operating either via inversion or retention of configuration, commonly via an S_N_2-type mechanism (Schober and Faber 2013): haloalkane and haloacid dehalogenases (Verschueren et al. 1993), epoxide hydrolases (Kotik et al. 2012), glycosidases (Lairson and Withers 2004) and sulfatases (Pogorevc and Faber 2002). The enzymes comprising the latter group are covered in this review with respect to their occurrence across all kingdoms of life, their substrate scope and the discovery of novel sulfatase enzymes.

**Scheme 1:**
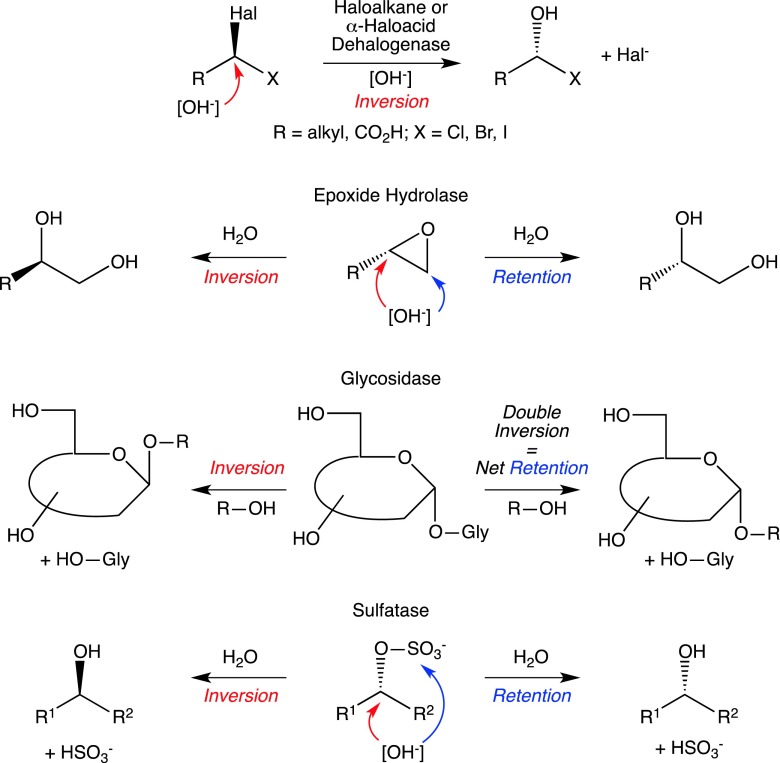
Stereochemical consequences of catalysis by retaining and inverting hydrolases, i.e., dehalogenases, epoxide hydrolases, glycosidases and sulfatases

Dehalogenases (EC 3.8.1.5) offer a wide variety of biotechnological applications and hence these enzymes were studied in great detail over the last decades. They are employed for the bioremediation of soils including the decontamination of chemical warfare agents (Fetzner 1998). For biocatalytic applications, they were exploited for the kinetic resolution of racemic *sec*-haloorganic compounds, such as haloalkanes and α-halocarboxylic acids to obtain valuable chiral building blocks for organic synthesis (Koudelakova et al. 2013; Janssen 2007; van Leeuwen et al. 2012). The majority of haloalkane and α-haloacid dehalogenases operate via an S_N_2 mechanism leading to inversion of configuration at the chiral carbon atom bearing the halide (Scheme 1). An aspartate residue within the active site acts as nucleophile, by replacing the halide to form a transient 'alkyl-enzyme intermediate'. The latter is hydrolytically cleaved by H_2_O, which is activated by a histidine residue thereby releasing the inverted product alcohol and liberating the Asp residue, which closes the catalytic cycle (Verschueren et al. 1993).

The enzymatic ring opening of oxiranes is achieved by epoxide hydrolases (EC 3.3.2.3), which serves as detoxification strategy to yield more innocuous diols (Kotik et al. 2012). Mechanistically, hydrolysis can occur either under retention or inversion of configuration (Scheme 1). If the water molecule attacks a substituted carbon atom, the stereochemical outcome is inversion of configuration. Retention is achieved by attack at an unsubstituted carbon yielding a retained diol. From detailed studies on epoxide hydrolase from *Aspergillus niger*, it was shown that nucleophilic attack is caused by an aspartate residue, which forms a 'glycol-monoester enzyme-intermediate'. Attack by a histidine-activated water molecule releases the diol (Widersten et al. 2010).

Another example for stereochemical alteration of a substrate is the enzyme class of glycosidases (EC 3.2.1.x) cleaving the glycosidic bonds. Depending on their mechanistic pathway, both inversion and retention of configuration are possible (Scheme 1). The former reaction is facilitated by a glutamate residue in the active site activating the nucleophile (ROH), which attacks the anomeric carbon atom through an S_N_2-type mechanism with inversion of configuration going in hand with release of the leaving group. Alternatively, glycoside cleavage may proceed via double inversion, leading to net retention. In this case, two glutamic acid residues are involved in catalysis. The first acts as acid via protonation of the anomeric oxygen forming a glycosyl oxonium species. In the second step, the other glutamate residue acts as base and deprotonates the nucleophile (ROH), which in turn attacks the enzyme-bound glycosyl species and releases the retained product (O'Hagan and Schmidberger 2010; Lairson and Withers 2004).

## Sulfatases

Due to the large number and broad diversity of sources, where sulfatases were obtained, they constitute a very heterogenic group of enzymes (Gadler and Faber 2007a; Hanson et al. 2004). So far, three distinct classes have been identified according to their substrate spectrum and mechanistic aspects (Kertesz 2000).

### Aryl sulfatases

**Fig. 1:**
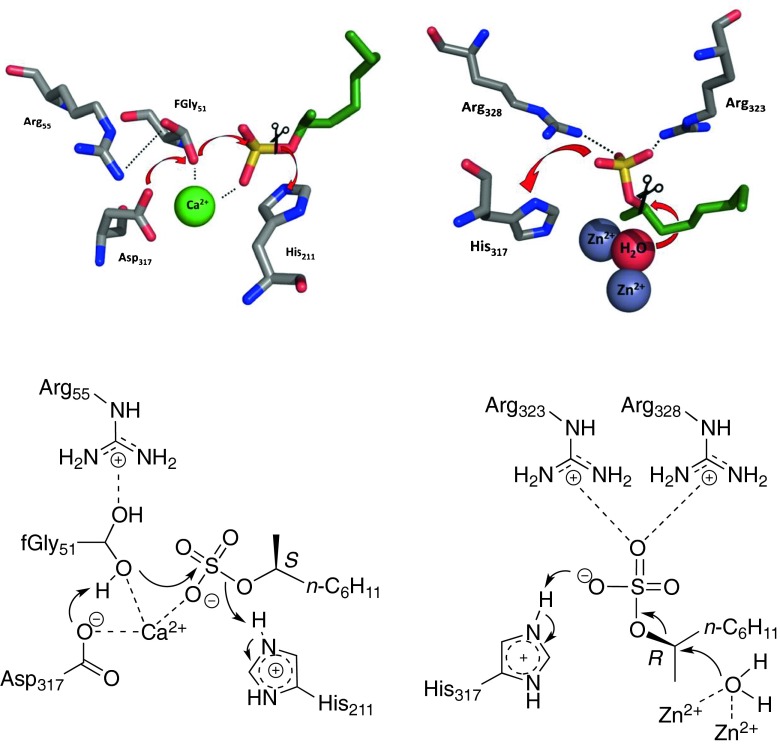
Catalytic residues and their mode of action for the retaining sulfatase PAS from *Pseudomonas aeruginosa* (PDB 1HDH, top left) and the inverting sulfatase Pisa1 from *P.* sp. DSM 6611 (PDB 4AXH, top right). Preferred enantiomers of the substrate 2-octyl sulfate (*green*) were docked into the active site using Schrödinger Maestro (Schrödinger Maestro Software Suite 2013) The flow of electrons implying nucleophilic attack is indicated by *red arrows*, and the S–O/C–O bonds being broken are marked by *scissor symbols*. The schematic mechanism is given below. Some amino acid residues were omitted for clarity. Pictures were generated using Pymol (Pymol Software 2013)

The name of the class is derived from the aromatic sulfate ester *p*-nitrophenyl sulfate, which is used as standard substrate for activity detection. However, many of them also act on sulfated carbohydrates, such as chondroitin sulfate and dermatan sulfate (Anson et al. 1992; Tomatsu et al. 1991). Due to their significance in humans, they have been studied in great detail with respect to their structure and mechanism and they clearly represent the best studied group of sulfatases. Fifteen of these enzymes have been identified in humans, which are linked to a lysosomal storage disease known as MSD (multiple sulfatase deficiency) (Hopwood and Ballabio 1999). Their function is to control the sulfation state of messenger molecules (Ahmed and James 1999). Several aryl sulfatases have been crystallised and structurally elucidated, including the human aryl sulfatase B (NP_000037) and the prokaryotic aryl sulfatase from *P. aeruginosa* (PA0183) (Fig. 1). The most notable aspect of aryl sulfatases is the highly conserved consensus motif C/S-X-P-X-A-X_4_-T-G (Kertesz 2000) which applies to all of the identified enzymes within this class, except *Rhodococcus ruber* sulfatase (G352_02444) (Gadler and Faber 2007b). The lead residue in this motif is either a cysteine or serine, which is post-translationally modified into a catalytically active aldehyde (hydrate) known as C_α_-formylglycine. This residue is unique to this class of enzymes and has not yet been observed in other active sites, except for phosphatases (Jonas et al. 2008). So far, cysteine is only found in eukaryotic sources, while bacterial hosts contain either a cysteine or a serine in the 'precursor' enzyme (Benjdia et al. 2010). Depending on the catalytic residue, cysteine-type sulfatases are typically found in the cytosol, whereas serine-type sulfatases are located in the periplasm (Cloves et al. 1977; Marquordt et al. 2003; Murooka et al. 1990). Due to the non-chiral nature of aryl sulfates, the stereochemical consequences of aryl sulfatase catalysis were not investigated.

### Fe^2+^-dependent sulfatases

The second class of sulfatases belong to the Fe^2+^-dependent dioxygenases, which catalyse an oxidative cleavage of the sulfate ester moiety to form the corresponding aldehyde and inorganic sulfate (Müller et al. 2004). In contrast to the other sulfatases, this reaction requires α-ketoglutarate as a redox-cosubstrate. The most prominent representative of this group is the alkyl sulfatase Atsk from *Pseudomonas putida* (PP4_02270) (Kahnert and Kertesz 2000). For biocatalytic applications, this class of sulfatases is less valuable, since a stereocenter is destroyed during the course of the reaction.

### Alkyl sulfatases

Inspired by the observation that the bacterium *Pseudomonas* sp. C12B (NCIB 11753) is able to grow on the common surfactant sodium dodecyl sulfate (SDS) in the 1960s (Payne et al. 1965; Williams and Payne 1964), sulfatase research turned into a hot topic due to potential applications in bioremediation. Initially, only little attention was paid to the stereochemical implications of sulfate ester hydrolysis, but investigations on *sec*-alkyl sulfate substrates revealed that there were two modes of operation involved in sulfate ester cleavage (Matcham et al. 1977). The retaining pathway cleaves the S–O ester bond, releasing the product alcohol without stereochemical alteration. In contrast, the inverting mechanism involves attack of an activated water molecule on the chiral carbon atom bearing the sulfate ester, thereby breaking the C–O bond and releasing the inverted alcohol (Fig. 1). While the retaining pathway was elucidated already in 2001 (Boltes et al. 2001), the inverting mechanism was elucidated only recently with the *sec-*alkyl sulfatase Pisa1 (FR850678) (Knaus et al. 2012). The catalytic mechanism of this β-lactamase type enzyme involves a binuclear Zn^2+^ cluster activating a water molecule which launches a nucleophilic attack on the chiral carbon atom. A histidine residue protonates the sulfate moiety, transforming it into a good leaving group (Knaus et al. 2012). The synthetic potential of this enzyme was demonstrated in a sulfatase-assisted total synthesis of the anti-leukemic agent (*R*)-Lasiodiplodin methyl ether (Fuchs et al. 2013). Further enzymes of this class include the *prim*-alkyl sulfatase SdsA1 (PA0740) (Hagelueken et al. 2006) and the *sec-*alkyl sulfatase SdsAP (HQ189533) (Long et al. 2011), both of which originate from *Pseudomonas* sp. and show high sequence similarity to Pisa1. To date, only limited structural information is available for β-lactamase-type alkyl sulfatases, i.e., Pisa1 and SdsA1.

## Search for sulfatase activity

### Discovery of novel sulfatases

Considering the vast amount of possible microbial sources for sulfatases, guidelines facilitating the search for novel sulfatase activities are desirable, which can be delineated from successful case stories in the literature.

**Scheme 2:**
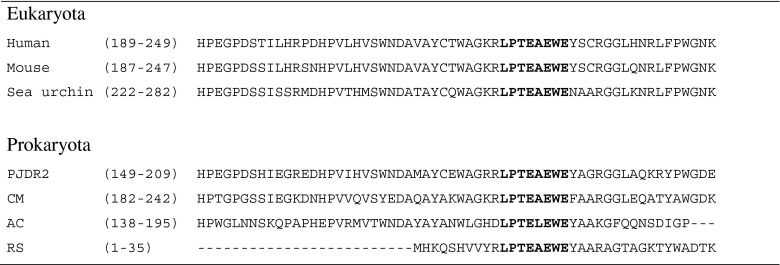
Partial sequence alignment of SUMF1 gene derived proteins. *PJDR2*, *Paenibacillus* sp. JDR-2 (YP_003009726); *CM*, *Cupriavidus metallidurans* (YP_586663); *AC*, *Acinetobacter calcoaceticus* (YP_004994666); *RS*, putative FGE-protein from *Ralstonia solanacearum* RFBP2957 (YP_003747422); human (NP_877437); mouse (NP_666049); sea urchin (XP_782973). Sequence alignment was done with clustal omega (Sievers et al. 2011). Numbers in brackets indicate the aligned amino acid residues. Letters highlighted in *bold* display the conserved sequence across Eukaryota and Prokaryota

The strongest indication for aryl sulfatase activity is probably the existence of sulfatase maturation enzymes, required for the post-translational modification of a cysteine or serine into the catalytically active C_α_-formylglycine aldehyde, or the corresponding catalytically active hydrate (Fig. 1). Given the uniqueness of this residue, it constitutes a strong marker for aryl sulfatase activity (Scheme 2).

Two systems are known to promote the maturation of Cys and/or Ser into the C_α_-formylglycine residue. The sulfatase maturation factor 1 (*SUMF1*) gene codes for the so called formylglycine-generating enzyme (FGE) (Dierks et al. 2003), which acts on Cys. The second system, termed anaerobic sulfatase maturating enzymes (anSME), was initially believed to be specific for serine-type sulfatases (Hanson et al. 2004). Recent studies, however, have shown that cysteine-type enzymes are also accepted via this *S*-adenosyl-l-methionine mediated pathway (Berteau et al. 2006). The genetic origin can be found within homologues of the *Klebsiella pneumoniae-*derived AtsA gene (AF262989_2) and has been investigated with the sulfatase from *Clostridium perfringens* ATCC 13124 (Q0TTH1) (Benjdia et al. 2007). However, due to limited sequence information, this approach is not always applicable.

### Metabolic demand for sulfur

As an essential element for growth, sulfur uptake occurs through various pathways, depending on the organism. Usually inorganic sulfate is available and is transformed through a cascade of reactions to yield the high-energy intermediate 3′-phosphoadenosine-5′-phosphosulfate (PAPS). Ultimately, sulfur is incorporated into the essential amino acids cysteine and methionine. In case inorganic sulfate is unavailable, organisms are forced to express other sulfur metabolizing enzymes, such as alkyl sulfatases, which are able to hydrolytically cleave inorganic sulfate from organic sulfate esters. The dependence of organisms on sulfate for growth opens a window for opportunities to induce sulfatases when sulfate esters are offered as the sole sulfur source.

Another and more general approach lies in the identification of a rich and robust (in)organic sulfate metabolism as a pointer for potential sulfatase activity, following the reasoning that the activity on (in)organic sulfate might be connected to alkyl sulfate esters and hence sulfatase activity. Sulfur metabolising organisms are typically found within the kingdom of Archaea due to their evolutionary background (Huber and Stetter 1998). This approach proved to be highly successful and led to the discovery of several highly selective sulfatase activities, such as from *Sulfolobus acidocaldarius* DSM 639 (Wallner et al. 2005b).

The most common indicator for sulfatase activity is the ability of organisms to grow on detergent-contaminated soil or wastewater and several bacteria have been isolated from such sources (Dodgson et al. 1954; Payne et al. 1965; Williams and Payne 1964). However, it has been shown that the reverse argument, i.e., the inability to degrade SDS should exclude sulfatase activity, cannot be drawn. A broad screening of bacteria and fungi by Gadler and Faber in 2007 for potential sulfatase activity on 2-octyl sulfate resulted in several highly active strains from *Pseudomonas* sp., together with many inactive candidates (Gadler and Faber 2007b). The most recent example is the *sec*-alkyl sulfatase Pisa 1 isolated from *Pseudomonas* sp. DSM 6611. Although this enzyme is inactive on SDS, it has a remarkably broad activity on a wide variety of *prim*- and *sec*-sulfate esters (Schober et al. 2012).

### Growth conditions

Forcing an organism to express the desired sulfatase activity is usually achieved by limiting the available sulfur to organically bound sources, such as short chain alkyl sulfates or (more common) the surfactant SDS. Since uptake of the preferred sulfur source (inorganic sulfate) (Kertesz and Wietek 2001) proceeds through different metabolic pathways, in comparison to organically bound sulfate (Cook et al. 1998; Lie et al. 1998; Marzluf 1994), supplementing the growth medium with sulfate esters drives the metabolism towards the desired direction. Importantly though, the chosen growth conditions should only be selective, but not growth limiting (Kertesz 2000). Under such constraints, organisms express sulfate starvation-induced (SSI) proteins. They usually include low sulfur containing enzymes, where cysteine and methionine in non-essential positions are substituted by other amino acids. *P. putida* strain KT2440, for example, is able to reduce the total soluble cellular thiol content 5-fold under sulfur limiting conditions (Scott et al. 2007).

Due to the fact that the expression of aryl sulfatases in several bacteria is strongly upregulated when sulfate is unavailable (Dodgson et al. 1982; Kertesz et al. 1993), it has been suggested that sulfatase enzymes are expressed at a lower level than other proteins (Kertesz 2000), which makes selective growth constraints all the more important to successfully obtain sulfate ester hydrolysing cells. Consequently, when the growth medium of *P. aeruginosa* IFO 3901 was supplemented with intermediates of the primary sulfur assimilation pathway, such as sufate, sulfite or cysteine, no enzyme expression was observed (Harada 1964). Conversely, supplementing the growth medium of *Coryneform* sp. B1a with phenyl sulfate allowed the organism to accept several primary alkyl sulfates as substrates (Matts et al. 1994).

It is noteworthy that some alkyl sulfatases are only expressed at certain stages of growth or in presence of different sulfur sources, which may complicate the screening and might lead to 'false negatives' in search for sulfatases.

Prime examples for this case are the *Pseudomonas* strains C12B (NCIB 11753) and AE-A. The C12B strain is probably the most thoroughly examined organism regarding sulfatase activity and harbours alkyl sulfatases P1 and P2. While P1 is constitutive and expressed onwards from the late exponential phase, the P2 enzyme is inducible and expressed only transiently during the exponential phase (Cloves et al. 1977, 1980; Ellis et al. 2002). A zymographic analysis of *Pseudomonas* strain AE-A showed up to three different alkyl sulfatases, depending on the nature of the supplemented sulfur source. In nutrient broth, only one sulfatase could be identified. With addition of SDS to the medium, a second alkyl sulfatase was observed, which migrated further towards the anode than the first enzyme. Finally, with the branched 2-butyl-1-octyl sulfate as supplement, a third alkyl sulfatase could be identified.

### Occurrence of microbial alkyl sulfatases

Several higher eukaryotes have been shown to harbour sulfatases, a few have even been put to use in technical applications, such as soil sulfate analysis using a sulfatase from *Helix pomatia* (AF109924) (Burgundy edible snail) (Whalen and Warman 1996). Others, like human sulfatases, are connected to the lysosomal storage disorder and are an important subject of biomedical studies (Hanson et al. 2004). These enzymes, however, originate from higher eukaryotic sources, which are usually not applicable to biotransformations. Besides the difficulties in obtaining substantial amounts of biomass in a reproducible way, post-translational modification, such as glycosylation or acetylation are additional but unavoidable challenges in the expression of active proteins. In contrast, prokaryotes, lower eukaryotes and several *Archaea* species are advantageous in terms of fast growth rates, facile enzyme cloning and heterologous expression and usually omit the need for cellular post-translational enzyme modification, which speeds up the screening for sulfatase-harbouring organisms. The following section gives an overview of microorganisms with proven sulfatase activities together with the respective substrate scope (Table 1).

**Table 1:** Classification of sulfate esters employed as test substrates for the screening of whole microbial cells and/or purified enzymes for sulfatase activity

				Substrate	Type					
	A		B		C		D		E	
	*R* ^1^	*R* ^2^	*R* ^1^	*R* ^2^	*R* ^1^	*R* ^2^	*R* ^1^	*R* ^2^	*R* ^1^	*R* ^2^
1	H	*n*-C_3_H_7_	CH_3_	*n*-C_3_H_7_	CH=CH_2_	*n*-C_4_H_9_	CH_3_	C≡C–C_2_H_5_	CH_3_	3,5-(CF_3_)_2_C_6_H_3_
2	H	*n*-C_4_H_9_	CH_3_	*n*-C_4_H_9_	CH=CH_2_	*n*-C_5_H_11_	CH_3_	CH_2_–C≡C–CH_3_	CH_3_	*p*-F-C_6_H_4_-CH_2_
3	H	*n*-C_5_H_11_	CH_3_	*n*-C_5_H_11_	CH=CH_2_	*n*-C_6_H_13_	CH_3_	C≡C–Ph	CH_3_	*p*-Cl-C_6_H_4_-CH_2_
4	H	*n*-C_6_H_13_	CH_3_	*n*-C_6_H_13_	CH=CH_2_	*n*-C_7_H_15_	C_2_H_5_	C≡C–CH_3_		
5	H	*n*-C_7_H_15_	CH_3_	*n*-C_7_H_15_	CH_3_	(CH_2_)_2_CH=C(CH_3_)_2_	C_2_H_5_	C≡C–C_2_H_5_		
6	H	*n*-C_8_H_17_	CH_3_	*n*-C_8_H_17_	CH_3_	CH_2_–CH=CH_2_	C≡CH	*n*-C_3_H_7_		
7	H	*n*-C_9_H_19_	CH_3_	*n*-C_9_H_19_	CH_3_	(CH_2_)_2_CH=CH_2_	C≡CH	CH_2_–CH(CH_2_)_3_		
8	H	*n*-C_11_H_23_	CH_3_	*n*-C_10_H_21_			C≡CH	*n*-C_4_H_9_		
9	H	*n*-C_12_H_25_	CH_3_	*n*-C_12_H_25_			C≡CH	*n*-C_5_H_11_		
10	H	*n*-C_13_H_27_	C_2_H_5_	C_2_H_5_						
11	H	*n*-C_14_H_29_	C_2_H_5_	*n*-C_4_H_9_						
12	SLES		C_2_H_5_	*n*-C_5_H_11_						
13	2-BOS		*n*-C_3_H_7_	*n*-C_3_H_7_						
14			*n*-C_3_H_7_	*n*-C_4_H_9_						
15			*n*-C_3_H_7_	*n*-C_6_H_13_						
16			*n*-C_4_H_9_	*n*-C_4_H_9_						
17			CH_3_	c-C_6_H_11_						
18			CH_3_	Ph						
19			CH_3_	CH_2_-Ph						
20			CH_3_	(CH_2_)_2_-Ph						

*SLES* sodium lauryl ether sulfate (Na^+^ *n*-C_12_H_25_–O–(CH_2_)_2_–O–SO_3_^−^), *2-BOS* 2-butyl-1-octyl sulfate

### Whole cell preparations

Whole cell preparations enable fast screening procedures and less work-up compared to the use of purified enzymes, which has to be paid for by reduced average activities. A comprehensive overview of microbial strains harbouring sulfatase activities is given in Table 2.

**Table 2:** Substrate scope, activities and enantioselectivities for sulfatase-activities of whole cell preparations

Organism	Substrate type	Conversion (%)^a^	*E* ^b^	Refs.
Prokaryota				
Proteobacteria				
*Pseudomonas* sp. C12B (NCIMB 11753)	A 4,5,6,8,9,10,11,12	n.d.	n.a.	(Gadler and Faber 2007b; Thomas and White 1991; Dodgson et al. 1982)
	B 3,4,5,12,14	<5–10	4–13	
	C 5	5–10	1	
*Pseudomonas* sp. DSM 6978	B 3,4,5,12,14	<5–10	4– > 200	(Gadler and Faber 2007b)
*Pseudomonas* sp. DSM 6611	B 3,4,5,9,14,18,20 C 5	5–21	6– > 200	(Gadler and Faber 2007b)
*Pseudomonas* sp. S7	A 9	n.d.	n.a.	(Yeldho et al. 2011)
*Pseudomonas* sp. AE-A	A 9	n.d.	n.a.	(Ellis et al. 2002)
*Pseudomonas* sp. AE-D	A 13	n.d.	n.a.	(Ellis et al. 2002)
*Pseudomonas* ATCC 19151	A 9	n.d.	n.a.	(Jovcic et al. 2010)
*Klebsiella oxytoca*	A 9	n.d.	n.a.	(Shukor et al. 2009)
*Comamonas* sp. DSM 115091	B 4	5–10	16	(Gadler and Faber 2007b)
*Citrobacter braakii*	A 9,12	n.d.	n.a.	(Dhouib et al. 2003)
*Rhizobiaceae* sp. FCC 175	B 4	>10	2	(Gadler and Faber 2007b)
*Cupriavidus necator* DSM 5536	B 4	<5	4	(Gadler and Faber 2007b)
*Paracoccus* sp. DSM 6392	B 3,4,12,14	<5–10	1–3	(Gadler et al. 2009)
	C 5	5–10	2– > 200^d^	
*Ralstonia eutropha* SPT0002 FCC120	B 4	>10	1	(Gadler and Faber 2007b)
*Ralstonia eutropha* sp. DSM 6428	B 4	5–10	21	(Gadler and Faber 2007b)
*Xanothbacter autotrophicus* DSM 431	B 4	>10	2	(Gadler and Faber 2007b)
*Xanothbacter autotrophicus* DSM 6696	B 4	<5	2	(Gadler and Faber 2007b)
*Xanothbacter flavus* DSM 338	B 4	<5	2	(Gadler and Faber 2007b)
*Xanothbacter flavus autotrophicus* DSM 3874	B 4	<5	7	(Gadler and Faber 2007b)
*Achromobacter* sp. FCC 175	B 4	<5	2	(Gadler and Faber 2007b)
Actinobacteria				
*Rhodococcus ruber* DSM 44541^e^	A 5,8	<5	n.a.	(Gadler and Faber 2007a)
	B 3,4,5,6,7,12,14,15,19 C 2	4–68	1–21	(Pogorevc and Faber 2002)
*Gulosibacter molinativorax* DSM 13485	B 4	>10	5	(Gadler and Faber 2007b)
*Nocardia nova* DSM 43843	B 4	>10		(Gadler and Faber 2007b)
Planctomycetes				
*Rhodopirellula baltica* DSM 10527	A 5	26	n.a.	(Wallner et al. 2005a)
	B 3,4,5,12,14 C 2,5	<5–18	2– > 200	
Cyanobacteria				
*Synechococcus* sp. PCC 7942	B 3,4,5,12,14	<5–24	1–3	(Gadler et al. 2009)
	C 5	5–10	4– > 200^d^	
*Synechococcus* sp. RCC 556	B 3,4,5,12,14	<5–10	n.d./1	(Gadler et al. 2009)
Firmicutes				
*Bacillus cereus*	A 9	n.d.	n.a.	(Singh et al. 1998)
*Bacillus sphaericus* FCC 098	B 4	<5	1	(Gadler and Faber 2007b)
Strain combination				
*Acinetobacter calcoaceticus* + *Pantoea agglomerans*	A 9	n.d.	n.a.	(Abboud et al. 2007)
Archaea				
Crenarchaeota				
*Sulfolobus acidocaldarius* DSM 639	B 3,4,5,12,14,19	10–43	5– > 200	(Gadler and Faber 2007a; Wallner et al. 2004)
	C 5	5–10	n.d.	
	E 2,3	5–10	2	
*Sulfolobus solfataricus* DSM 1617	B 4,19	20–56	2–35	(Wallner et al. 2005b)
*Sulfolobus shibatae* DSM 5389	B 4,19	20–43	2–48	(Wallner et al. 2005b)
*Sulfolobus metallicus* DSM 6482	B 4	5–10	1	(Wallner et al. 2005b)
*Sulfolobus hakoniensis* DSM 7519	B 4	5–10	1	(Wallner et al. 2005b)
*Acidianus brierley* DSM 1651	B 4	5–10	1	(Wallner et al. 2005b)
*Acidianus infernus* DSM 3191	B 4	25	1	(Wallner et al. 2005b)
*Acidianus ambivalens* DSM 3772	B 4	13	1	(Wallner et al. 2005b)
*Metallosphaera sedula* DSM 5348	B 4	5–10	1	(Wallner et al. 2005b)
*Sulfurisphaera ohwakuensis* DSM 12421	B 4	11	1	(Wallner et al. 2005b)

*n.a.* not applicable, *n.d.* not determined

^a^In kinetic resolutions showing high enantioselectivity, the maximum conversion is 50 %

^b^Enantioselectivity is expressed as the ratio of the reaction rate of enantiomers ('enantiomeric ratio', *E*) (Straathof and Jongejan 1997)

^c^Unpublished results

^d^Improved *E* values in presence of organic cosolvents

^e^Crude enzyme preparation

### Prokaryotic strains

Prokaryotes encompass the largest group among all potential sulfatase harbouring organisms. In addition to their abundance, they can be easily accessed under simple growth conditions. Several strains possessing the ability to degrade sulfate-based detergents have recently been identified, which indicates the existence of sulfatases. The enantioselectivities — expressed as 'enantiomeric ratio' (*E*) (Straathof and Jongejan 1997) — range from virtually nil (*E* ~ 1) to perfect (*E* > 200) depending on the strains.

The largest phylum is occupied by proteobactera. In particular, *Pseudomonas* strains have been positively identified for their alkyl sulfatase activity. Newly discovered strains include *Citrobacter braakii* and a previously unknown *Pseudomonas* strain MTC 10311. Even a combination of strains — *Acinetobacter calcoaecticus* and *Pantoea agglomerans* — was successfully employed. *C. braakii* (strain CTM 50600) is able to degrade sodium lauryl ether sulfate over the course of 6 h starting from a concentration of 0.6 g l^−1^ and the bacterium also exhibited growth up to an OD_600_ of 1.0 with SDS as a sole carbon source (Dhouib et al. 2003). These properties indicate the occurrence of sulfatases which enable the cells to use also the carbon moiety of the detergent for growth. A BLAST search with the (limited) available sequence information from *C. braakii* strain CTM 50600 on the basis of the protein sequences of the sulfatases from *P. aeruginosa* and Pisa1 did not reveal any positive hits.

The aforementioned *Pseudomonas* species have shown to harbour sulfatases which were thoroughly characterised and which exhibited a broad applicability for biocatalytic processes (Gadler and Faber 2007a,b; Schober et al. 2012). Another promising candidate is the strain *P. aeruginosa* MTC 10311. This bacterium was recently isolated from detergent contaminated soil and could degrade 1.4 g of SDS within 48 h. It also showed activity at 40 °C with a depletion capacity of 90 % compared to the initial amount of surfactant at this temperature (Ambily and Jisha 2012).

Interestingly, some strains only show degrading properties when they are embedded within a consortium of other bacteria, such as *Acinetobacter calcoaceticus* and *Pantoea agglomerans*. Neither *A. calcoaceticus* nor *P. agglomerans* alone was able to degrade SDS, whereas the combination of both bacteria resulted in 60 % degradation within 50 h and complete degradation within 130 h (Abboud et al. 2007).

### Eukaryotic strains

In comparison to prokaryotic sources for sulfatases, activities in eukaryotes are more scarce. Lower eukaryotic organisms, such as the green algae *Volvox carteri* is a potential hosts for alkyl sulfatase activity (Hallmann and Sumper 1994; Selmer et al. 1996). Higher eukaryotes, such as the Burgundy edible snail *Helix pomatia* contains sulfatases, whose genes have been identified and cloned (Wittstock et al. 2000), and which were found to be active on aryl sulfates (Whalen and Warman 1996; Yegles et al. 1997). Hence, they were also tested with several alkyl sulfates to investigate latent activity towards this substrate class. Moderate activity was obtained towards *prim-*alkyl sulfates but unfortunately no conversion was observed when *sec-*alkyl sulfatases were employed (Schober 2013).

A sulfatase from *V. carteri f. nagariensis* (strain HK10) could be purified to homogeneity and was assayed with aryl sulfates, such as *p-*nitrophenyl sulfate, 4-nitrocatechol sulfate and 5-bromo-4-chloro-3-indolyl sulfate and activity was detected for all of those compounds. In contrast, the alkyl sulfate SDS was not converted (Hallmann and Sumper 1994).

### Sulfatases from *Archaea*

Owing to the importance of sulfur metabolism during the first billion of years of life, the third domain — Archaea — complement a particularly rich source of sulfatases. These hyperthermophilic organisms prefer a strongly acidic environment (pH 2–3) and temperatures ranging between 55 °C and 95 °C, which is well above the usual 37 °C (Wallner et al. 2005b). On the premise of harbouring a robust sulfur metabolism, several hyperthermophilic strains were examined in search for novel alkyl sulfatase activities by Wallner et. al. in 2005 (Wallner et al. 2005b). The occurrence of sulfatase activity was strongly influenced by the growth conditions: While cells could be obtained under availability of oxygen, no growth was observed in the absence of O_2_. Optimisation in terms of the carbon source revealed that sucrose was most beneficial. Among the tested species, *Sulfolobus acidocaldarius* DSM 639 turned out to be most promising. This strain was able to convert both linear and branched *sec*-alkyl sulfate substrates with low to good conversion and (occasionally) also excellent enantioselectivity (*E* > 200) (Wallner et al. 2004).

## Purified sulfatases

Compared to the data available for whole cell preparations, only very few purified sulfatase enzymes have been characterised with respect to their substrate tolerance towards alkyl sulfates (Table 3). Since whole cells displaying low enantioselectivity most likely contain multiple sulfatases with different (or even opposite) enantiopreference, which makes protein isolation tedious and complicated, the prime target for the isolation of novel sulfatases were strains showing excellent stereoselectivites, because they are (most likely) expected to contain only a single sulfatase, which is easier to identify on the protein level.

**Table 3:** Substrate scope, activities and enantioselectivities for purified sulfatases

Kingdom	Substrate type	Conversion^a^ (%)	*E* ^b^	References
Prokaryota				
Pisa1	A 5	n.d.	n.a.	(Schober et al. 2011, 2012, 2013)
	B 1,2,4,6,11,12,14,18	5–57	10– > 200	
	C 1,2,3,4,5,6,7	5–50	17– > 200^c^	
	D 1,2,3,4,5,6,7,8,9	5–57	8– > 200	
	E 1	49	>200	
PAS	D 3,4,5,7,8,9	46–65	2– > 200	(Schober et al. 2013)
	E 1	30	>200	
SdsA1	A 3,5,7,9	n.d.	n.a.	(Knaus et al. 2012; Hagelueken et al. 2006)
	B 4^d^	n.d.	n.d.	
SdsAP	A 9	n.d.	n.a.	(Long et al. 2011)
*Comamonas terrigena* CS1	B 4	n.d.	n.d.	(Gadler and Faber 2007a)
*Comamonas terrigena* CS2	B 3,4,5,6,8,9	n.d.	n.d.	(Gadler and Faber 2007a)
*Rhodococcus ruber* S2	B 4	n.d.	21– > 200^e^	(Gadler and Faber 2007a)
*Pseudomonas* S1	B 3,4,6	n.d.	n.d.	(Gadler and Faber 2007a)
*Pseudomonas* S2	B 4	n.d.	n.d.	(Gadler and Faber 2007a)
*Pseudomonas* S3	B 4,10,13,16	n.d.	n.d.	(Gadler and Faber 2007a)
*Coryneform* sp. B1a	A 1,2,3,4,5	n.d.	n.a.	(Matts et al. 1994)
*Aerobacter aerogenes* ^f^	A 6	n.d.	n.a.	(Schober 2013)
Eukaryota				
*Helix pomatia* ^f^	A 6	n.d.	n.a.	(Schober 2013)
	D 9	n.d.	n.d.	

*n.a.* not applicable, *n.d.* not determined

^a^In kinetic resolutions showing high enantioselectivity, the maximum conversion is 50 %

^b^Enantioselectivity is expressed as the ratio of the reaction rate of enantiomers ('enantiomeric ratio', *E*) (Straathof and Jongejan 1997)

^c^Improved *E* values in presence of organic cosolvents

^d^Only the (*R*)-enantiomer of B4 was tested

^e^In the presence of Fe^3+^

^f^Commercially available

Considering the source of purified enzymes, the majority of isolated sulfatases so far were derived from prokaryotic sources, with few exceptions, such as *Helix pomatia* aryl sulfatase (Thies 1979). To date, no enzyme could be purified from Archaea, despite their promising activity towards alkyl sulfates (Wallner et al. 2004, 2005b).

With Pisa1, PAS, SdsA1, *Pseudomonas* S1-3 (NCIB 11753) and SdsAP, the majority of purified sulfatases were derived from the phylum of Proteobacteria, which is not suprising, given the fact that these strains ranked among those exhibiting the highest *E* values and activities observed in whole cell screenings. The *Comamonas terrigena* enzymes S1 and S2 also accepted several *sec-*alkyl sulfates; however, no quantitative data are available regarding their enantioselectivity (Barrett et al. 1980). The enzyme from *Coryneform* sp. B1a accepted several primary alkyl sulfate esters with chain lengths ranging from three to seven carbon atoms. *Aerobacter aerogenes* ATCC 9621 and *Helix pomatia* sulfatases are commercially available and have been tested in our laboratory for their respective substrate scopes; however, they only showed a very limited substrate spectrum in comparison to other sulfatases, since they were only active on *prim*-sulfate esters but did not accept *sec*-sulfates (Schober et al. 2013).

### Selectivity enhancement and immobilisation

When using whole cell systems or partially purified enzyme preparations, insufficient enantioselectivites are often encountered, which most likely are due to the existence of competing multiple sulfatases possessing different (or even opposite) stereoselectivities. In order to circumvent this drawback, several methods for the selectivity enhancement of alkyl sulfatase reactions were developed.

## Cosolvents

The most convenient technique is the use of organic cosolvents, which have been applied to purified enzymes and whole cell biocatalysts alike. For the purified enzyme Pisa1 from *Pseudomonas* sp. DSM 6611, a thorough study for a range of common polar and apolar organic solvents was conducted. Among them, DMSO was found to suppress background hydrolysis of sulfate esters bearing activated allylic and benzylic functional groups. The latter increased *E* values significantly. Enhanced enantioselectivities were also observed for whole cell preparations of cyanobacteria, such as *Synechococcus elongatus* PCC 7942 and *Paracoccus denitrificans* DSM 6392 in combination with lower alcohols, such as MeOH, EtOH or *t-*ButOH. *E* values could be improved up to >200 for both strains, albeit at the expense of decreased conversion (Gadler et al. 2009).

## Metal ions

Several aryl and alkyl sulfatases depend on metal ions required for catalysis. Aryl sulfatases, for example the enzyme PAS from *P. aeruginosa,* relies on Ca^2+^ for proper orientation and binding of the negatively charged substrate (Boltes et al. 2001), whereas the alkyl sulfatase Pisa 1 from *Pseudomonas* sp. DSM 6611 needs two Zn^2+^ ions for water activation to provide a good nucleophile [OH^−^] for the hydrolytic cleavage. Supplementation with other metal ions, such as Fe^2+^ and Fe^3+^, were found to have a dramatic effect on the enatioselectivity of the *Rhodococcus ruber* DSM 44541 RS2 enzyme. In presence of 5 mM of FeCl_3_, the selectivity could be improved from an E value of 3.6 to >200 (Pogorevc et al. 2002).

## Immobilisation

Reusability and recyclability are important features of biocatalysts to make them industrially more appealing. At present, only few sulfatase preparations have been subjected to immobilisation. First successful experiments were conducted with immobilised cells of *Pseudomonas* C12B on polyacrylamide beads, which resulted in similar activities towards primary alkyl sulfate esters compared to the free enzyme. Total degradation of sulfate esters was observed within 48 h and an encouraging residual activity of 13 % was retained after 13 days of use (Thomas and White 1991). Further whole cell experiments with *Pseudomonas* C12B and *Comamonas terrigena* N3H were performed on various supports to evaluate their applicability as biofilms in wastewater treatment plants (Roig et al. 1998a, b). While *C. terrigena* N3H did not exhibit activity towards SDS, *Pseudomonas* C12B, which is a well known SDS-degrader (Dodgson et al. 1982), was also able hydrolyse the surfactant in immobilized form (Roig et al. 1998a; Thomas and White 1990).

In 2002 Pogorevc et al. were able to bind a crude preparation of the *Rhodococcus ruber* DSM 44541 RS2 enzyme onto a DEAE- and Ecteola-cellulose carrier resulting in ~100 % and 22 % residual activity, respectively (Pogorevc et al. 2002). Further studies in this direction would certainly lead to more widespread biotechnological applications of sulfatases, given the fact that several new sulfatases have emerged since the initial immobilization studies were conducted.

Although the phylogenetic relationship of organisms harbouring alkyl sulfatases is very heterogenic, the majority of enzymes identified and characterised on a molecular level derive from prokaryotic sources. So far, a broad range of more than fifty different *prim*- and *sec*-alkyl sulfate esters tested as substrates were successfully converted to the corresponding alcohol with inversion or retention of configuration, depending on the source and nature of the enzyme. Although there are several crystal structures available, mostly of the class of aryl sulfatases, sequence and structural information are still scarce with regard to alkyl sulfatases. Given the rapid advances in molecular biology, the number of alkyl sulfatases is expected to increase significantly in the near future, which will broaden the applicability of these useful hydrolytic enzymes in the design of enantioconvergent processes for the generation of single stereoisomeric *sec*-alcohols from the corresponding *rac-sec*-sulfate esters (Schober and Faber 2013; Schober et al. 2013).
